# Biochemical and functional characterization of SpdA, a 2′, 3′cyclic nucleotide phosphodiesterase from *Sinorhizobium meliloti*

**DOI:** 10.1186/1471-2180-13-268

**Published:** 2013-11-26

**Authors:** Céline Mathieu-Demazière, Véréna Poinsot, Catherine Masson-Boivin, Anne-Marie Garnerone, Jacques Batut

**Affiliations:** 1 INRA, Laboratoire des Interactions Plantes-Microorganismes (LIPM), UMR441, F-31326 Castanet-Tolosan, France; 2 CNRS, Laboratoire des Interactions Plantes-Microorganismes (LIPM), UMR2594, F-31326 Castanet-Tolosan, France; 3 Laboratoire IMRCP UMR UPS/CNRS 5623, Université Paul Sabatier, Toulouse, Cedex 31062, France

**Keywords:** *Sinorhizobium*, 3′, 5′cAMP, 2′, 3′cAMP, Phosphodiesterase, RNA degradation, Crp

## Abstract

**Background:**

3′, 5′cAMP signaling in *Sinorhizobium meliloti* was recently shown to contribute to the autoregulation of legume infection. *In planta*, three adenylate cyclases CyaD1, CyaD2 and CyaK, synthesizing 3′, 5′cAMP, together with the Crp-like transcriptional regulator Clr and *smc02178*, a gene of unknown function, are involved in controlling plant infection.

**Results:**

Here we report on the characterization of a gene (*smc02179, spdA*) at the *cyaD1* locus that we predicted to encode a class III cytoplasmic phosphodiesterase.

First, we have shown that *spdA* had a similar pattern of expression as *smc02178 in planta* but did not require *clr* nor 3′, 5′cAMP for expression.

Second, biochemical characterization of the purified SpdA protein showed that, contrary to expectation, it had no detectable activity against 3′, 5′cAMP and, instead, high activity against the positional isomers 2′, 3′cAMP and 2′, 3′cGMP.

Third, we provide direct experimental evidence that the purified Clr protein was able to bind both 2′, 3′cAMP and 3′, 5′cAMP *in vitro* at high concentration. We further showed that Clr is a 3′, 5′cAMP-dependent DNA-binding protein and identified a DNA-binding motif to which Clr binds. In contrast, 2′, 3′cAMP was unable to promote Clr specific-binding to DNA and activate *smc02178* target gene expression *ex planta*.

Fourth, we have shown a negative impact of exogenous 2′, 3′cAMP on 3′, 5′cAMP-mediated signaling *in vivo*. A *spdA* null mutant was also partially affected in 3′, 5′cAMP signaling.

**Conclusions:**

SpdA is a nodule-expressed 2′, 3′ specific phosphodiesterase whose biological function remains elusive. Circumstantial evidence suggests that SpdA may contribute insulating 3′, 5′cAMP-based signaling from 2′, 3′ cyclic nucleotides of metabolic origin.

## Background

*Sinorhizobium meliloti* is a soil-born α-proteobacterium that can enter a nitrogen-fixing symbiosis with *Medicago sativa* (alfalfa) and related legumes. The establishment of the symbiosis relies on a complex molecular dialogue between the two partners that triggers two essential and overlapping steps, nodulation and infection (see [1,2] for reviews). During the infection process, bacteria colonize root hairs forming Infection Threads (ITs) that extend and proliferate towards the nodule primordium that is formed in the root cortex. Ultimately, rhizobia are released from ITs within nodule cells where they fix molecular dinitrogen. Nodulation and infection are tightly controlled processes and we have shown recently that bacterial adenylate cyclases (ACs) contribute to the negative autoregulation of infection [3].

ACs (EC 4.6.1.1) are enzymes that synthesize cAMP (3′, 5′-cyclic adenosine monophosphate) from ATP. There are 6 non-homologous classes of ACs as a typical example of convergent evolution [4,5]. Class III is the universal class whose members can be found in both prokaryotes and eukaryotes although, to our knowledge, their presence in plants has not been established [6]. The number of class III ACs strikingly varies in bacteria. *E. coli* has none whereas cyanobacteria, mycobacteria and rhizobia, a group of phylogenetically-diverse bacteria [7], have many, up to 32 in the soybean symbiont *Bradyrhizobium japonicum*. The biological function of class III ACs in bacteria remains poorly understood. Class III ACs synthesize cAMP in response to environmental cues such as light, oxygen, nitrogen and pH in Cyanobacteria [8] or high osmotic pressure in *Myxococcus xanthus*[9,10]. Class III ACs are also involved in biotic interactions as they contribute to virulence in *M. tuberculosis*, *P. aeruginosa* and in some fungal pathogens [5,11-13]. CO_2_ and Ca^2+^ are signals used by pathogens to sense their host environment through their AC–cAMP signaling systems. *Candida albicans* and mycobacteria express CO_2_-responsive ACs [5,14] whereas CyaB from *P. aeruginosa* is Ca^2+^ sensitive. Another example of cAMP-associated signal being used by the human fungal pathogen *C. albicans* to sense the host environment is the bacterial peptidoglycan present in blood serum [15].

We have recently described the first instance of class III ACs contributing to a symbiotic (mutualistic) interaction, between *Sinorhizobium meliloti* and its host plant *Medicago sativa*[3]. *S. meliloti* has 26 class III ACs of overall unknown biological functions with a variety of domain organization [16]. In response to a plant signal present in nodules, three receptor-like adenylate cyclases CyaD1, CyaD2 and CyaK synthesize the secondary messenger molecule 3′, 5′cAMP. 3′, 5′cAMP together with the Crp-like transcriptional activator Clr in turn promote transcription of the target gene *smc02178*, of unknown biochemical function [3]. We have recently found that this cascade contributes to the autoregulation of the symbiotic interaction. Specifically, activation of the cAMP cascade in nodules inhibits, by a mechanism that remains to be elucidated, secondary infection by rhizospheric bacteria. This control is lost in either a triple *cyaD1cyaD2cyaK* mutant, a *clr* or a *smc02178* mutant resulting in a hyper-infection phenotype on plants–*ie* an abundance of abortive ITs on roots–as a consequence of a relaxed control of secondary infection [3].

The concentration of the second messenger 3′, 5′cAMP in cells is controlled at the level of its synthesis by ACs and/or by its degradation to 5′AMP by phosphodiesterases (PDEs). PDEs are a superfamily of enzymes divided in three, non-homologous, main classes. All mammalian PDEs as well as several enzymes identified in *Drosophila*, *Caenorhabditis* and *Saccharomyces cerevisiae* belong to class I, whose conserved carboxy-terminal catalytic domain contains two invariant motifs H(X)_3_H(X)_25-35_D/E [17]. Class II PDEs are enzymes from *Saccharomyces cerevisiae*, *Dictyostelium discoideum*, *Schizosaccharomyces pombe*, *C. albicans*, and *Vibrio fischeri*[17]. This class of enzymes shares the conserved motif HXHLDH. Class III PDEs belong to the superfamily of metallophosphoesterases [18]. They share the conserved sequence motif D-(X)_n_-GD(X)_n_-GNH[E/D]-(X)_n_-H-(X)_n_-GHXH as well as a βαβαβ secondary structure signature [17].

Here we report on the characterization of a class III PDE from *S. meliloti* (SpdA, SMc02179) that we anticipated from the localization of the *spdA* gene at the *cyaD1* locus to be involved in signal termination by turning-over the secondary messenger 3′, 5′cAMP. We have found that purified SpdA had actually no detectable activity against 3′, 5′cAMP and, instead, had high activity on the structural isomer 2′, 3′cAMP, which may occur in cells as a by-product of RNA degradation [19]. We demonstrated that, contrary to 3′, 5′cAMP that promoted Clr binding to a cognate binding-site, 2′, 3′cAMP bound unproductively to Clr. Although SpdA biological function remains to be established, we present circumstantial evidence that SpdA may insulate 3′, 5′cAMP-mediated signaling from 2′, 3′-structural isomers.

## Results

### SpdA, a putative PDE

**Figure 1:**
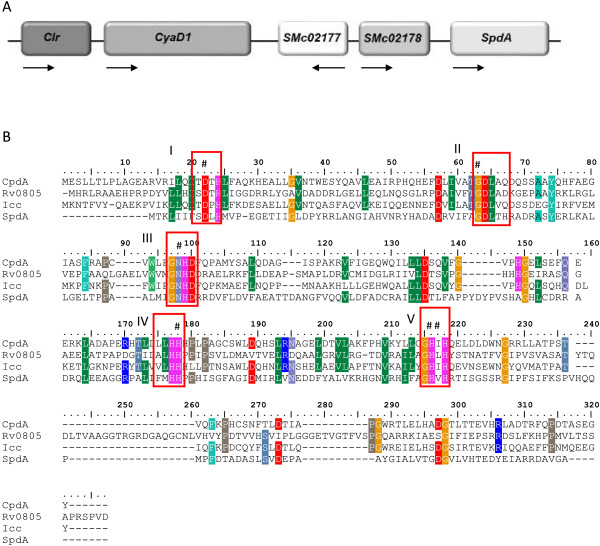
**SpdA, a putative phosphodiesterase at the *cyaD1 *locus. (A)** Genetic map of the *cyaD1* locus on the *S. meliloti* chromosome. Arrows indicate the direction of transcription. **(B)** SpdA has the five conserved subdomains (boxed) of class III phosphodiesterases. Sequence alignment of SpdA with cyclic adenosine monophosphate phosphodiesterases from *Escherichia coli* (CpdA), *Mycobacterium tuberculosis* (Rv0805) and *Haemophilus influenzae* (Icc) and *S. meliloti*. The invariant amino acids forming the metal ion binding sites of class III PDEs are marked with (#). Alignment was made using ClustalW algorithm [23].

Inspection of the *cyaD1* locus (Figure 1A), that contains the *clr* gene as well as the *clr*–target gene *smc02178*, pointed to the *smc02179* gene product as a potential PDE that we subsequently coined SpdA. SpdA belongs to a 15-member protein family sharing the IPR004843 domain characteristic of a wide range of metallophosphoesterases, among which phosphorine phosphatases, nucleotidases, and class III PDEs. We thus compared SpdA as well as the 14 other IPR004843-containing proteins to known PDEs from *Mycobacterium tuberculosis* (Rv0805), *Haemophilus influenzae* (Icc) and *Escherichia coli* (CpdA and CpdB) [20-22].

Overall analysis of the whole protein family indicated no clear phylogenetic relationship between the family members besides the fact that SMc04449 and SMc04018 behaved as an outgroup together with CpdB, a periplasmic 2′, 3′ cAMP-PDE from *E. coli* (see Additional file 1). SpdA closest homologue was *M. tuberculosis* Rv0805 and indeed closer sequence inspection indicated that SpdA contained the 5 sub-domains characteristic of Rv0805 and other class III PDEs [17] (Figure 1B) whereas all other *S. meliloti* proteins, except SMc02712, had fewer (see Additional file 1). SpdA had a predicted cytoplasmic location and missed the amino-terminal 200-aminoacid membrane anchoring domain of Rv0805 [24].

### *spdA* is expressed *in planta*, independently of *clr* and 3’, 5’cAMP

**Figure 2:**
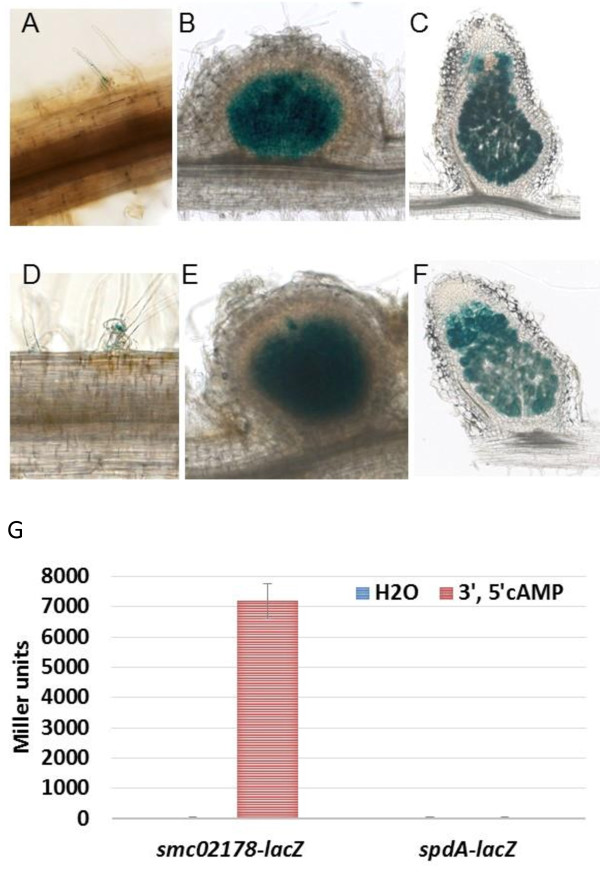
**SpdA is expressed *in planta*, independently of *clr*.** Expression of a *spdA-lacZ* reporter gene fusion in *S. meliloti* 1021 **[A-C]** and *clr* mutant **[D-F]**, in infection threads **(A, D)**, young nodules (7 dpi) **(B, E)** and mature nodules (14 dpi) **(C, F)** of *M. sativa*. **(G) ***spdA-lacZ* expression was monitored *ex planta* in *S. meliloti* 1021 strain after addition of 5 mM 3′, 5′cAMP or water as a negative control. *smc02178-lacZ* was used as a control.

We probed expression of a translational *spdA-lacZ* fusion (pGD2179, See Additional file 2) that contained the intergenic region between *smc02178* and *spdA* (Figure 1A) as well as the first 12 codons of *spdA*. The *spdA-lacZ* fusion did not detectably express *ex planta* and instead expressed in *Medicago sativa* nodules with the same pattern as *smc02178*[3]*i.e.* expression in young nodule primordia and in zones II and III of mature nodules (Figure 2A-F). However, *spdA* expression *in planta* was independent of *clr*, and *ex planta* expression could not be induced by exogenous 3′, 5′cAMP, in contrast to *smc02178* expression (Figure 2G). None of the environmental conditions or compounds which we have tested was able to stimulate *spdA* expression *ex planta,* including 3′, 5′cGMP, 2′, 3′cAMP, 5′AMP, nodule extracts, root exudates or several growth and stress conditions (See Additional file 3).

Altogether these results indicated that *spdA* was expressed *in planta* from its own promoter and had the same expression pattern as *smc02178* although the two genes were not co-regulated.

### SpdA is a 2′, 3′cNMP PDE

**Figure 3:**
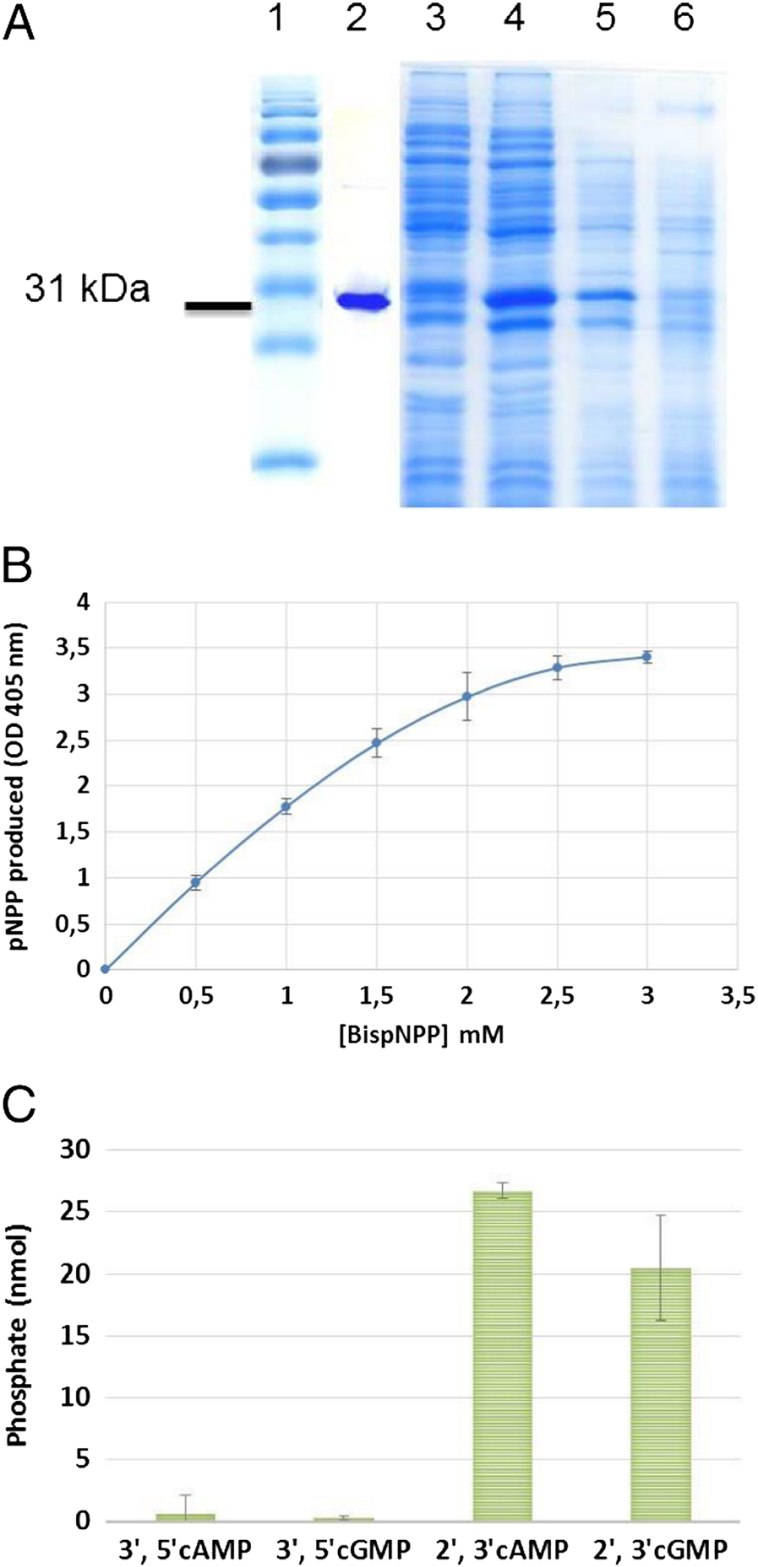
**SpdA is a phosphodiesterase. (A)** Purification of SpdA-His_6_ protein on a Ni agarose column (Qiagen). 1: Molecular weight markers, 2: Purified SpdA-His_6_, 3: culture sonication supernatant, 4: Column flowthrough, 5: *E. coli* BL21(DE3) pET::2179 cells treated with IPTG, 6: *E. coli* BL21(DE3) pET::2179 cells, no IPTG. **(B)** SpdA was incubated with the general phosphodiesterase substrate bis-pNPP. The amount of *p*-nitrophenol produced was measured at 405 nm. **(C)** Phosphodiesterase activity was measured from phosphate release after incubation of cyclic nucleotides with SpdA and CIP.

We purified the SpdA protein as a carboxy-terminal His_6_-tagged fusion (Figure 3A). Under non-denaturing electrophoretic conditions the protein migrated as a monomer. Purified His_6_-SpdA protein displayed activity against the generic PDE substrate BispNPP *in vitro* (Figure 3B). SpdA had little or no activity against either 3′, 5′cAMP or 3′, 5′cGMP but significantly hydrolyzed the positional isomers 2′, 3′cAMP and 2′, 3′cGMP (Figure 3C) which are products of RNA degradation [19]. The Km for 2′, 3′cAMP was 3.7 mM and kCat was 2 s^-1^ indicating a slow enzyme with low affinity for its substrate *in vitro* (See Additional file 4). We observed no inhibition of the enzyme by its substrate and found that 3′, 5′cAMP did not affect SpdA activity on 2′, 3′cAMP.

Despite IPR004843-containing proteins being documented metalloenzymes, the metal chelators EDTA, 1-10-Phenanthroline and Bipyridyl, or the addition of Fe^2+^ or Mn^2+^ metal ions, had no effect on SpdA activity (see Additional file 5). Mass spectrometry of isolated SpdA confirmed the absence of associated metal including Mg^2+^, Mn^2+^ and Co^2+^ together with the monomeric state of the protein. Indeed, a well resolved single mass peak corresponding to the monomer was observed after Max-Ent deconvolution of the spectra.

### 2′, 3′cAMP binds unproductively to Clr

**Figure 4:**
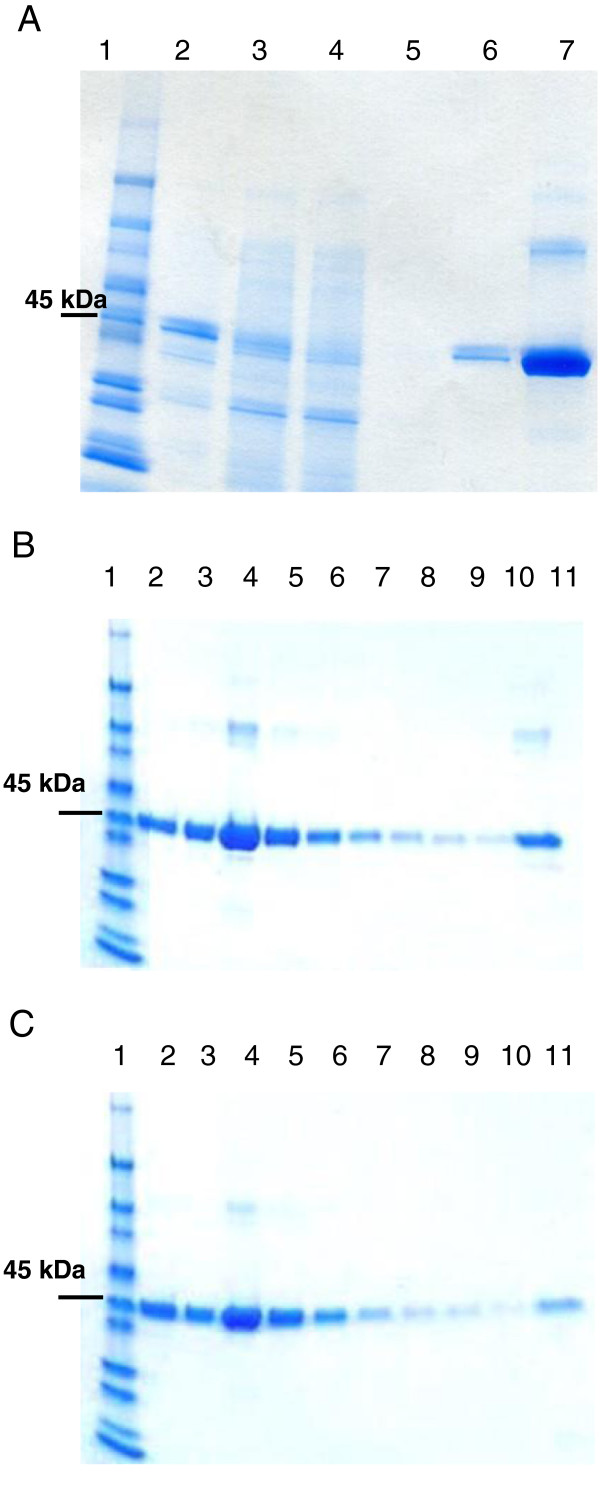
**Purified Clr binds 3′, 5′cAMP and 2′, 3′cAMP nucleotides *in vitro. *(A)** Clr-GST purification on a glutathione sepharose column. 1: Molecular weight markers, 2: Bacterial sonication pellet, 3: Sonication supernatant, 4: Column flowthrough, 5: Column wash, 6: Purified Clr-GST, 7: Clr-GST concentrated on centricon CO10000. **(B,C)** Clr affinity chromatography on a 3′, 5′cAMP-Agarose column (Sigma) and fraction analysis by SDS-PAGE (4-12%). 1: Molecular weight markers, 2: Free Clr (load), 3 : Flowthrough, 4-10: column wash, 11: eluted fraction by either 30 mM 3′, 5′cAMP **(B)** or 30 mM 2′, 3′cAMP **(C)**.

In order to investigate a possible interference of 2′, 3′cyclic nucleotides with 3′, 5′ cAMP-signaling we assessed the capacity of 2′, 3′cAMP and 3′, 5′cAMP to bind Clr *in vitro*. For this purpose, we purified a GST-tagged version of Clr by affinity purification (Figure 4A). Purified Clr protein was loaded onto a 3′, 5′cAMP-agarose column. Bound Clr protein was then eluted with either the cognate 3′, 5′cAMP nucleotide or its 2′, 3′ isomer (30 mM). Both nucleotides displaced agarose-bound Clr thus suggesting that Clr could bind 3′, 5′cAMP and 2′, 3′cAMP at the same binding site (Figure 4B, C).

**Figure 5:**
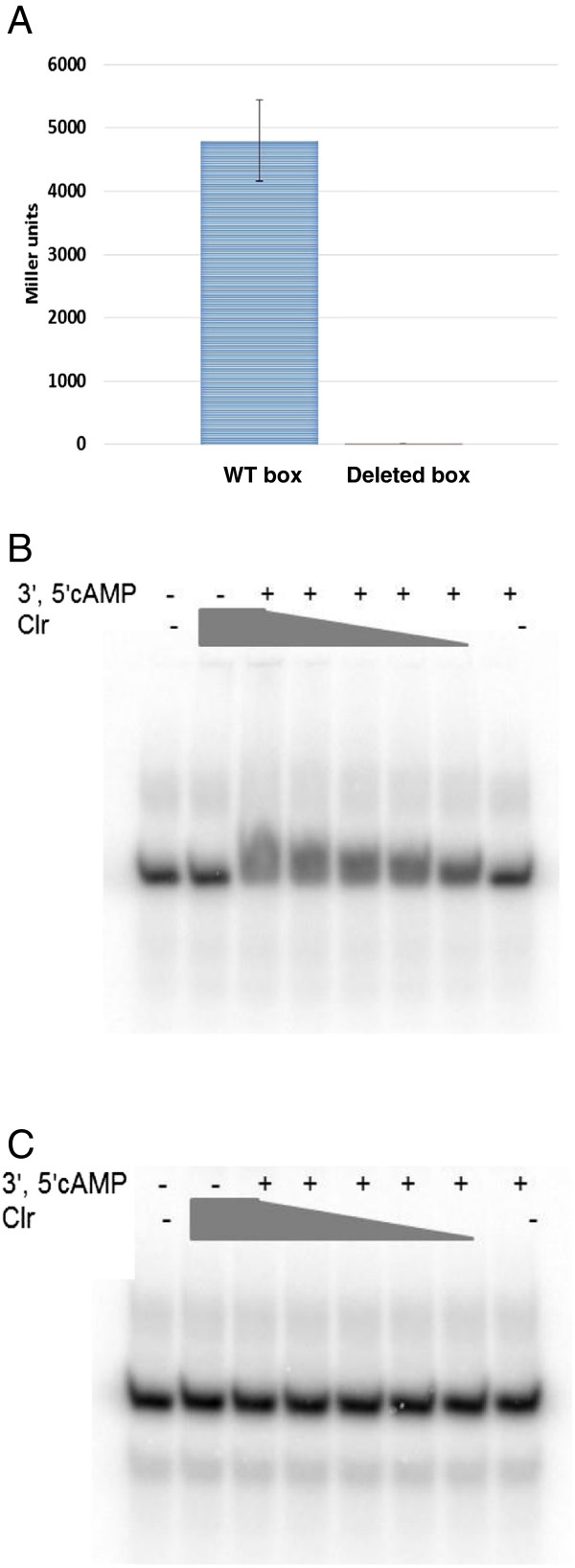
**3′, 5′cAMP promotes Clr binding to the Clr-box at the *smc02178 *promoter. (A) ***smc02178-lacZ* expression was monitored *ex planta* in *S. meliloti* 1021 WT and a Clr-box deleted strain (**TG**Δ**CA**) after addition of 3′, 5′cAMP. **(B, C)** EMSA assays showing Clr-GST binding to 28-mers oligomers carrying the WT Clr-box **(B)** or a mutated version **(C)** (see Additional file 10). Assays were performed in the presence of 1.75 nM oligomers, 200 μM 3′, 5′cAMP, and varied amounts of Clr (35 μM, 17.5 μM, 8.75 μM, 3.5 μM and 1.75 μM). See methods for details.

**Figure 6:**
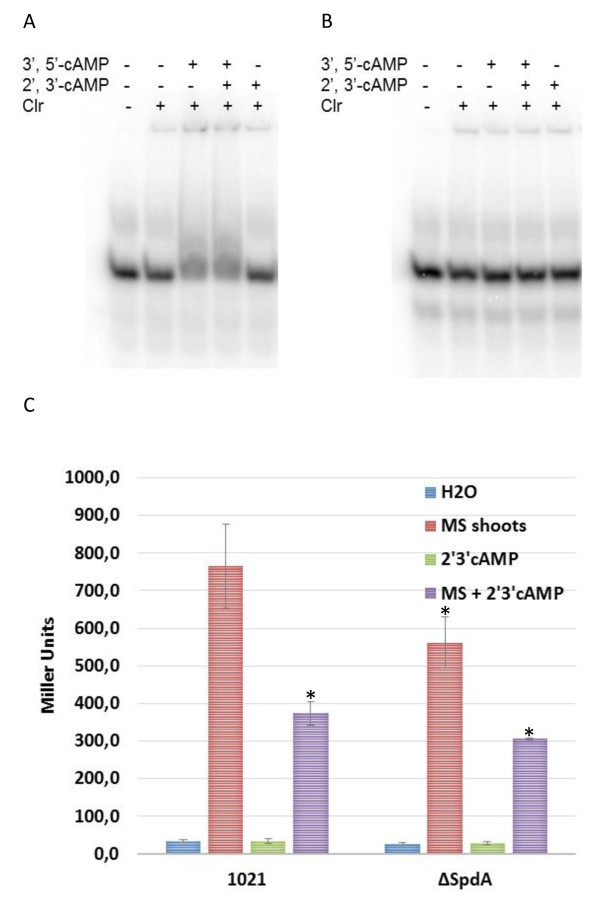
**2′, 3**′**cAMP effect on Clr-DNA binding and *smc02178 *expression. (A, B)** EMSA assays showing Clr binding to 28-mers oligomers including the wt Clr-box **(A)** or a mutated version **(B)**, as in Figure 5. Assays were performed in the presence of 1.75 nM oligomers, 200 μM 3′, 5′cAMP and/or 200 μM 2′, 3′cAMP, and 69 μM Clr (for details, see methods). **(C) ***smc02178-lacZ* expression was monitored *ex planta* in *S. meliloti* 1021 WT and a ΔSpdA strain after addition of *M. sativa* shoots extract (MS) and/or 7.5 mM 2′, 3′cAMP. *p < 0.03 compared to the wild type.

Clr is a predicted transcriptional activator of the Crp family [3]. Inspection of the *smc02178* promoter region pointed to a short palindromic sequence (**TGTT**CCGCGGGA**AACA**) centered *ca*. 68 bp upstream of the predicted start codon that was a potential binding site for Clr. Accordingly, deletion of this motif abolished activation of the *smc02178* promoter by *clr* in the presence of exogenously provided 3′, 5′cAMP (Figure 5A). In order to directly assess whether this motif was a binding site for the Clr protein, we tested the ability of purified Clr-GST to bind DNA oligomers (28-mers) bracketing the putative Clr-binding motif (Figure 5B) or a mutated version (Figure 5C). We found that Clr induced a retard in oligomer migration that was strictly dependent on the presence of 3′, 5′cAMP, of an intact Clr-box and was Clr concentration-dependent. However, no clear shifted band was observed, irrespectively of the binding and gel electrophoresis conditions tested, which probably reflected dissociation of the Clr/cAMP/DNA complex. Nevertheless we interpreted this as evidence that Clr bound the predicted Clr-box in a 3′, 5′cAMP-dependent manner. 2′, 3′cAMP was unable to promote Clr binding to the Clr-box, at the same concentration as 3′, 5′cAMP. Mixed incubation of the two nucleotides (1/1) with Clr *in vitro* showed no detectable effect of 2′, 3′cAMP on DNA-binding by Clr (Figure 6A, B).

We tested the impact of exogenously provided 2′, 3′cAMP on *smc02178* expression *in vivo* under different experimental conditions. Exogenous 2′, 3′cAMP alone was unable to promote activation of the *smc02178-lacZ* reporter fusion *in vivo,* even at high (7.5 mM) concentration (Figure 6C). In contrast 2′, 3′cAMP had a negative impact on 3′, 5′cAMP-driven *smc02178* expression. Inhibition reached 50% (Figure 6C) when 3′, 5′cAMP was produced endogenously, as in normal physiological conditions, upon addition to the bacterial culture of a *Medicago* shoot extract containing the plant signal that triggers activity of the CyaD1CyaD2CyaK ACs [3]. Inhibition was only 30% when 3′, 5′ cAMP was provided exogenously (See Additional file 6). Noteworthy, the negative impact of 2′, 3′cAMP was not observed on a constitutive *hemA-lacZ* reporter fusion (pXLGD4, see Additional file 2 and Additional file 6) suggesting a specific effect of 2′, 3′cAMP on 3′, 5′cAMP-mediated signaling.

### Biological characterization of a *S. meliloti spdA* null mutant

As to get an insight into SpdA biological function we inactivated the corresponding gene by cre-lox deletion [25]. *spdA* inactivation decreased *smc02178-lacZ* expression by *ca.* 25% in the presence of plant shoot extracts, supposedly by increasing endogenous 2′, 3′cNMP concentration *in vivo.* Combining *spdA* inactivation together with exogenous 2′, 3′cAMP addition decreased *smc02178* expression to 40% of wild-type (Figure 6C and See Additional file 6).

The *spdA* mutant had the same growth characteristics as wild-type both in rich complex medium (LBMC) and in synthetic Vincent medium with mannitol and glutamate (VGM) as carbon and nitrogen sources (see Additional file 7). We observed that exogenous 2′, 3′cAMP extended bacterial growth in VGM medium, suggesting that *S. meliloti* can grow by utilizing 2′, 3′cAMP, as *Yersinia* does [26]. However the *spdA* mutant did not differ from wild-type in this respect. The *spdA* mutant also responded similarly to wild-type to various stress conditions including detergent (SDS) and heat shock (See Additional file 7).

*spdA* inactivation had no detectable effect on symbiotic performances, including nodulation, infection and nitrogen fixation (plant dry weight), on *Medicago sativa* nor on the level or pattern of *smc02178* symbiotic expression *in planta* (See Additional file 8).

Hence we did not detect any phenotype associated with the *spdA* mutation besides its limited effect on 3’, 5’ cAMP-signaling.

## Discussion

### Clr is a 3′, 5′cNMP-dependent DNA-binding transcriptional activator

The findings reported here give experimental support and extend the model proposed by [3], as we demonstrated that Clr binds to the *smc02178* promoter region at a specific site in a 3′, 5′cAMP-dependent manner. The transcription start site (TSS) at the *smc02178* promoter was not determined experimentally here. However a single *smc02178* TSS was mapped in the closely related strain 2011 by RNA-sequencing of a pool of bacteria living in 16 different free-living and stress conditions [27]. The TSS mapped 61.5 bp downstream of the center of the Clr-box which is the distance typically found in class I Crp(CAP)-dependent promoters. In Class I promoters, a single protein–protein interaction with CAP facilitates the binding of RNA-Polymerase to the promoter to yield the RNA-Polymerase–promoter closed complex [28].

One salient feature of Clr binding at the *smc02178* promoter DNA was instability. In spite of the many binding and electrophoresis conditions tested, we consistently observed a smear instead of a clear-cut band shift upon binding of Clr to its target DNA. One feature that may account for this instability is that the Clr binding site is **TGTT**N_8_**AACA**, a shorter palindrome as compared to the consensus *E. coli* CRP(CAP)-binding site **TGTGA**N_6_**TCACA**. Identification of this binding motif, together with transcriptome analysis experiments, will help identification of new Clr targets in the *S. meliloti* genome.

The reason for which 2′, 3′cAMP did not promote DNA-binding of Clr is unclear. Although Clr bound 2′, 3′cAMP *in vitro* at high concentration (30 mM), it may not do so at the concentration of 2′, 3′cAMP that we used in EMSA assays (200 μM). Alternatively, 2′, 3′cAMP may not trigger the appropriate conformational change that allows Crp binding to DNA. Further experiments are needed to distinguish between these two possibilities.

### SpdA encodes a 2′, 3′cNMP phosphodiesterase

Class III PDEs are metallophosphoesterases carrying the IPR004843 domain. IPR004843-containing proteins have a wide range of substrates, including cyclic nucleotides, and ensure a variety of biological functions [17]. *S. meliloti* has 15 uncharacterized IPR004843-containing proteins (see Additional file 1) and we have demonstrated that purified SpdA has a PDE activity *in vitro* (Figure 3).

We have further found that SpdA had no or little activity against 3′, 5′cAMP or 3′, 5′cGMP and instead had high activity against 2′, 3′cAMP or 2′, 3′cGMP. Although this cannot be formally excluded it is unlikely that SpdA would have a predominant 3′, 5′cAMP PDE activity *in vivo* since a SpdA null mutant had lower, and not enhanced, *smc02178* expression in vivo (Figure 6C).

Substrate specificity varies widely among class III PDEs. CpdA from *E. coli* and *P. aeruginosa*, Icc from *Haemophilus influenzae* are 3′, 5′cNMP PDEs [21,22,29] whereas *E. coli* CpdB was the first described 2′, 3′cNMP-specific PDE [30]. Rv0805 from *M. tuberculosis*, although it was first reported as a 3′, 5′cNMP PDE [20], has a much stronger activity (150 times fold) against 2′, 3′cNMP than against 3′, 5′cNMP [31]. *Myxococcus xanthus* PdeA and PdeB instead hydrolyse 2′, 3′cNMP and 3′, 5′cNMP with the same affinity [32]. Hence class III PDEs substrate specificity cannot be predicted from simple primary sequence inspection. It is thus possible that several IPR004843 proteins of *S. meliloti* display a 2′, 3′cyclic phosphodiesterase activity, thus contributing a functional redundancy.

A surprising feature of SpdA was the absence of associated metal ion which is, to our knowledge, unique among IPR004843-containing proteins. Rv0805 activity for example was not inhibited by metal chelators but was boosted by Mn^2+^ addition [20]. However, it has been already reported that the iPGM protein from castor bean that belongs to a superfamily of metalloenzymes [33] was actually metal-independent [34]. Moreover, the Carboxy-terminal HD domain of the *E. coli* tRNA nucleotidyltransferase has a metal-independent phosphodiesterase activity toward 2′, 3′cAMP [35]. Thus, the fact that SpdA displays metal-independent 2′, 3′cNMP-phosphodiesterase activity is not completely unprecedented. Mass spectrometric measurements performed under mild ionization conditions also pointed out that the well-defined monomeric form of the protein did not present any demetallation.

The 2′, 3′cNMP substrate specificity of SpdA leaves the question of 3′, 5′cAMP turnover intact. One option would be to identify a 3′, 5′cNMP PDE among the 14 other *S. meliloti* proteins containing the IPR004843 domain. Another, non-exclusive, possibility would be a regulation of 3′, 5′cAMP homeostasis by secretion rather than by degradation [36].

### Possible biological functions for SpdA

Very little is known about the origin, role and fate of 2′, 3′cyclic nucleotides. One documented origin is RNA degradation and physiological or stressful conditions may indeed lead 2′, 3′cNMPs to accumulate in bacteria. We are not aware of any other origin such as, for example, isomerization of corresponding 3’, 5’ cyclic nucleotides. In this context, SpdA may serve at least three different, non-exclusive, functions: a metabolic function, a detoxifying function and a role in preventing cross talk with 3′, 5′cAMP signaling.

Although *S. meliloti* likely metabolized exogenous 2′, 3′cAMP (See Additional file 7), *spdA* was not critical for this since the mutant grew indistinctly from wild-type under these conditions.

2′, 3′cAMP was recently reported to be a toxic compound in kidney cells, that opens mitochondria permeability transition pores thus leading to a pre-apoptotic and necrotic stage [37]. We thus considered whether SpdA may counteract a toxic effect of 2′, 3′cNMPs in *S. meliloti*. However the unaltered growth characteristics of the *spdA* mutant as compared to wild-type in various growth (including the presence of exogenous 2′, 3′cAMP) and stress conditions (see Additional file 7) did not give support to this possibility.

A third possibility would be SpdA preventing cross-talk between 2′, 3′cyclic nucleotides and 3′, 5′cAMP signaling. Several lines of evidence are in favor of this possibility: (i) the evolutionary-conserved physical location of *spdA* in close proximity to *cyaD1*, *clr* and the target gene *smc02178* in all the sequenced strains of *Sinorhizobium meliloti*, *Sinorhizobium saheli* and *Sinorhizobium fredii* (https://www.genoscope.cns.fr/agc/microscope/mage/); [38] (ii) *spdA* expression in nodules at the same place where 3′, 5′cAMP signaling takes places [3] and where a massive RNA degradation occurs as part of the reorientation of the bacteroid transcriptome to the goal of nitrogen fixation [39] (iii) a significant and specific decrease in *smc02178* expression upon providing exogenous 2′, 3′cAMP (iv) the spurious interaction of 2′, 3′cAMP with Clr.

Whatever SpdA function, the high Km value measured *in vitro* for the 2′, 3′cAMP substrate (3.7 mM) would imply that the cyclic nucleotide accumulates in high amounts in bacteroids, unless specific physiological or biochemical conditions lower Km value *in vivo*. Developing methods for direct measurements of 2′, 3′cNMP levels in bacteroids, where *spdA* preferentially expresses, is now needed to clarify this issue. A ribonucleic origin for 2′, 3′cAMP/cGMP would make sense physiologically given the extensive transcriptome reprofiling taking place in bacteroids [39] and the abundance of VapC-type ribonucleases in *S. meliloti* genome [40]. Intriguingly, the human intracellular pathogen *M. tuberculosis* shares with *S. meliloti,* despite the large phylogenetic distance separating them, a wealth of ACs, a Clr-like transcriptional regulator as well as a close homolog of SpdA, Rv0805. Rv0805, like SpdA, has a preferential activity–and similar Km value-towards 2′, 3′ cyclic nucleotides [31] and contributes to overall bacterial virulence on macrophages, by a still obscure mechanism [11,12,24]. Interestingly, *M. tuberculosis* and *S. meliloti* have in commun a high number of VapC-type RNases of the VapC(B)-toxin (antitoxin) family [40,41]. Altogether this suggests the intriguing possibility that SpdA, Rv0805 and other cytoplasmic PDEs may constitute a physiological adaptation in bacteria with a high RNA turnover, possibly in relationship with 3′, 5′cAMP-mediated signaling.

## Conclusion

Signal transduction in bacteria is dominated by two-component regulatory systems [42]. However, some bacteria, including important pathogens and symbionts, use cyclic or dicyclic nucleotide signaling for modulating interaction with their abiotic or biotic environment [43,44]. Characterization of enzymes and mechanisms that synthesize and degrade secondary messenger molecules, restrict their diffusion within the cell and prevent cross-talking by diffusible isomers, is needed for fully understanding cyclic nucleotide signaling. In the context of characterizing 3′, 5′cAMP-mediated signaling in the *S. meliloti*-*Medicago* symbiosis, we have identified a plant-expressed 2′, 3′cAMP/cGMP specific phosphodiesterase whose biological function remains to be elucidated. Circumstantial evidence suggests that one SpdA function could be to insulate 3′, 5′cAMP-based signaling from 2′, 3′ cyclic nucleotides of metabolic origin.

## Methods

### Bacterial strains, plasmids, and growth conditions

Plasmids and bacterial strains used in this study are listed in Additional file 2 and Additional file 9 respectively. *S. meliloti* strains were grown at 28°C in rich LB medium supplemented with 2.5 mM CaCl_2_ and 2.5 mM MgSO_4_ (LBMC) or in modified Vincent synthetic medium with glutamate (0.1%) and mannitol (1%) as nitrogen and carbon sources, respectively (VGM) [45]. *E. coli* strains were grown at 37°C in rich LB medium.

The concentrations of antibiotics used for *S. meliloti* cultures were 200 μg/ml for streptomycin, 100 μg/ml for neomycin, 10 μg/ml for tetracycline, and 30 μg/ml for gentamicin. The concentrations of antibiotic used for *E. coli* cultures were 50 μg/ml for ampicillin and 25 μg/ml for kanamycin.

### Stress responses

Bacterial response to SDS and heat shock was evaluated by analysis of the growth curves of WT and ΔSpdA mutant in liquid LBMC. Strains were challenged with SDS (0.01% v/v) at OD_600_ 0.1 and heat shock (50°C for 20 min) was applied to overnight cultures before dilution at OD_600_ 0.1. Aliquots were collected at different time intervals, OD_600_ was measured and residual growth was determined [46].

### Construction of plasmids and mutant strains

Primers used for DNA amplification are listed in Additional file 10. *S. meliloti* 1021 was used as template for DNA amplification. For deletion of the *spdA* gene, we used the cre-lox system [25]. PCR fragments encompassing the upstream/amino-terminal coding region and the downstream/carboxyl-terminal coding region of *spdA* were amplified using CreLox 2179 up Left-CreLox 2179 up Right and 2179 Down NcoI-2179 Down HincII as primers (See Additional file 10), digested by SacI-SacII and NcoI-HincII, and cloned into the SacI-SacII and NcoI-HincII restriction sites of pCM351, respectively. The resulting plasmid was introduced into the *S. meliloti* 1021 strain by conjugation. Transconjugants sensitive to tetracycline and resistant to gentamicin were screened. A *ΔspdA* mutant was selected. The *spdA*-expressing construct pET::2179 was obtained after amplification of the *spdA* gene-coding region using *S. meliloti* 1021 genomic DNA as template and LNdeI2179 and RHindIII 2179 as primers. The PCR fragment was digested with NdeI and HindIII and cloned into the NdeI-HindIII digested pET-22b plasmid to yield pET::2179. The *Clr*-expressing construct pGEX::clr was obtained after amplification of the *clr* gene-coding region using *S. meliloti* 1021 genomic DNA as template and ClrBamHI and ClrEcoRI as primers. The PCR fragment was digested with BamHI and EcoRI and cloned into the BamHI-EcoRI digested pGEX-2T to yield pGEX::clr.

To construct pGD2179, that carries a *spdA-lacZ* translational fusion, a 177-bp PCR fragment encompassing the *spdA* promoter region was amplified using 2179left and 2179right primers, digested with HindIII and BamHI, and cloned in the in-frame orientation at the same sites of the *lacZ* translational fusion plasmid pGD926. The pAMG2178 plasmid was obtained after amplification of the *smc02178* promoter-coding region using *S. meliloti* 1021 genomic DNA as template and BamHI 2178 and Hind BoxL as primers. For pAMG2178ΔClrbox, PCR fragments encompassing the upstream region Clr box and the downstream region Clr box of the *smc02178* promoter were amplified using 2178 H-BoxLPstI and X 2178-BoxRPstI as primers. The two fragments obtained were digested by PstI and then ligated and amplified by PCR using BamHI 2178 and Hind BoxL as primers. The two fragments p2178WT (134 bp) and p2178ΔClrbox (128 bp) were then cloned into pGD926 vector.

All constructs were verified by PCR and Sanger sequencing in *E. coli* and by PCR in *S. meliloti.* Plasmids were transferred from *E. coli* to *S. meliloti* by triparental mating using pRK600 as the helper plasmid. pET::2179 and pGEX::clr were directly transferred into *E. coli* BL21(DE3) and SP850 respectively.

### Protein purifications

For His_6_-SpdA purification, an overnight culture of *E. coli* strain BL21(DE3) pET::2179 expressing wild-type *S. meliloti spdA* was diluted at OD_600_ 0.1 in 250 ml of LB medium containing Ampicillin (Amp 50 μg/ml). Cultures were grown with shaking at 28°C. When the OD_600_ reached 0.8, 1 mM isopropyl β-D-1-thiogalactopyranoside (IPTG) was added, and cultures were grown for 5 additional hours. Bacteria were collected by centrifugation (10,000x g for 30 min at 4°C), and pellets were washed with 60 ml Tris buffer (20 mM Tris–HCl [pH 8.0]). Bacteria were collected by centrifugation (10,000x g for 30 min at 4°C), and pellets were stored at−80°C. All of the subsequent procedures were performed at 4°C. Thawed bacteria were resuspended in 5 ml of buffer A (50 mM Tris–HCl [pH 8.0], 250 mM NaCl, 10% glycerol) and lysed by sonication. The lysates were centrifuged to remove the cell debris at 10,000x g for 30 min at 4°C. The supernatant was loaded to a Ni-NTA resin (Qiagen) equilibrated with buffer B (50 mM Tris–HCl [pH 8.0], 250 mM NaCl, 10% glycerol, 10 mM Imidazol, and 5 mM *β-*Mercaptoethanol). After washing with the buffer B containing 20 mM Imidazol, the bound protein was eluted using the buffer B containing 250 mM Imidazol. Protein was desalted into buffer A. Purified protein aliquots were stored at−80°C.

For Clr-GST purification, an overnight culture of *E. coli* strain SP850 pGEX::clr expressing wild-type *S. meliloti clr* was diluted at OD_600_ 0.1 in 1 l of LB medium containing Ampicillin (Amp 50 μg/ml) and Kanamycin (Kan 25 μg/ml). Cultures were grown with shaking at 28°C. When the OD_600_ reached 0.8, 1 mM isopropyl β-D-1-thiogalactopyranoside (IPTG) was added, and cultures were grown for 5 additional hours. Bacteria were collected by centrifugation (10,000x g for 30 min at 4°C), and pellets were washed with 60 ml PBS buffer (140 mM NaCl, 2.7 mM KCl, 10 mM Na_2_HPO_4_, 1.8 mM KH_2_PO_4_, [pH 7.3]). Bacteria were collected by centrifugation (10,000x g for 30 min at 4°C), and pellets were stored at−80°C. All of the subsequent procedures were performed at 4°C. Thawed bacteria were resuspended in 10 ml PBS buffer and lysed by sonication. The lysates were centrifuged to remove the cell debris at 10,000x g for 30 min at 4°C. The supernatant was loaded to a Glutathione sepharose 4B resin (GE Healthcare) equilibrated with PBS buffer. After washing with PBS buffer, the bound protein was eluted using 50 mM Tris–HCl buffer [pH 8.0] containing 10 mM reduced glutathione. Protein was desalted on Amicon CO 10,000 (Millipore) and buffer exchanged with 0.1 M Phosphate buffer [pH 7.0] containing 50 mM NaCl. Purified protein aliquots containing 10% glycerol were stored at−80°C.

### Infusion ESI-Q-TOF experiment

ElectroSpray Ionization coupled with a quadrupole-time of flight tandem was used in the positive ion mode using a Q-TOF Ultima Instrument (Waters). The SpdA protein was dissolved in water with 0.05% formic acid and directly introduced into the source at a flow rate of 5 μl/min. Capillary entrance voltage was set to 2.7 kV, and dry gas temperature to 150°C. Voltages: Cone: 80 V, Rf lens: 40 V. MS profile [500 (10%), 1500 (60%), 2500 (20%), ramp 10%]. Scanning domain: m/z 1000-3000. Calibration was performed with orthophosphoric clusters. Continuum spectra exhibiting multicharged ions were transformed into molecular mass envelops using the MaxEnt 1 software.

### Electromobility shift assay

A set of DNA probes covering the predicted Clr binding palindrome were obtained by annealing two complementary oligonucleotides. The annealing reactions were performed in water with 25 μM strand + (WTN8+ or MN8+ (see Additional file 10)) and 25 μM strand–(WTN8-or MN8-(see Additional file 10)) for each probe in a total reaction volume of 100 μl. Mixes were incubated at 95°C during 5 min following by slow cooling to 25°C. 175 nM double-strands probes were end labelled using 20 μCi of [ATPγ-^32^P] and 10 U of T4 polynucleotide kinase (Promega). Probes (1.75 nM each) were incubated in binding buffer (10 mM Tris [pH 8.0], 1 mM EDTA, 1 mM DTT, 10 μg/ml bovine serum albumin, 100 mM KCl) containing 50 μg/ml poly(2′-deoxyinosinic-2′-deoxycytidylic acid) (Sigma) and 10% glycerol for 30 min at room temperature with purified Clr and 3′, 5′cAMP or 2′, 3′cAMP added to the concentrations indicated in the figure legends in a final reaction volume of 15 μl. Samples were subjected to electrophoresis on a 10% polyacrylamide TBE 0.5 X gel containing 4% PEG-8000. Electrophoresis was conducted in TBE 0.5 X buffer at 80 V at room temperature. Gels were dried and analysed by autoradiography.

### Plant assays and plant extracts preparation

Seeds of *M. sativa* cv. Europe were surface sterilized, germinated, and allowed to grow in 12-cm^2^ plates containing slanting nitrogen-free Fahraeus agar medium for 3 days at 22°C with day/night cycles of 16/8 h. The plants were inoculated with 2.10^3^ bacteria per plant. Nodules were counted every day during 8 days then every 2 days until 35 days post-inoculation (dpi). At 35 dpi, shoots were collected and dried overnight at 65°C for weight measurements. Plant extracts were prepared as previously described [3].

### β-Galactosidase assays

*S. meliloti* strains carrying the pGD2178, pXLGD4 or pGD2179 plasmids were grown at 28°C in VGM. Overnight cultures were diluted to an OD_600_ of 0.1 in VGM and grown for an additional 2 h. 5 ml-cultures supplemented with 3′, 5′cAMP (2.5 mM or 5 mM), 2′, 3′cAMP (7.5 mM) or 5 mM 3′, 5′cGMP were grown for an additional 5 hours at 28°C. Overnight incubation was used for other potential inducers listed in Additional file 3. β-Galactosidase activities were measured (Miller units) using 1 ml of culture (or 0.1 ml for overnight cultures), as previously described [47].

### Cytological techniques

Plants were inoculated with *S. meliloti* strains carrying the pGD2178 or the pGD2179 plasmid. Entire roots were collected 7 dpi or 14 dpi, fixed with 2% (vol/vol) glutaraldehyde solution for 1.5 h under vacuum, rinsed three times in Z buffer (0.1 M potassium phosphate buffer [pH 7.4], 1 mM MgSO_4_, and 10 mM KCl), and stained overnight at 28°C in Z buffer containing 0.08% 5-bromo-4-chloro-3-indolyl-D-galactoside (X-gal), 5 mM K_3_Fe(CN)_6_, and 5 mM K_4_Fe(CN)_6_. Nodules were harvested at 14 dpi, fixed with 2% (v/v) glutaraldehyde in Z buffer, and then sliced into 70 μm-thick longitudinal sections using a vibrating-blade microtome (VT1000S; Leica) before staining overnight at 28°C. Entire roots or nodule sections were observed under a light microscope.

### Phosphodiesterase activity assays

Biochemical assays were performed in 50 mM Tris–HCl [pH 8], 5 mM *β-*Mercaptoethanol, 10 mM NaCl, 100 μM MnCl_2_, and 0 to 2.5 mM bis-*P*-nitrophenyl phosphate in a total volume of 50 μl. Reactions were initiated by the addition of 120 nM SpdA and the reaction was stopped after 10 min at 25°C by the addition of 10 μl of 200 mM NaOH. Release of *p*-nitrophenol was determined by measuring the absorbance at 405 nm. Cyclic NMP assays were performed in reaction mixtures containing 50 mM Tris–HCl [pH 8], 5 mM *β-*Mercaptoethanol, 10 mM NaCl, 10 mM cyclic nucleotides, 1 μM SpdA and 10 U calf intestine phosphatase (CIP) were incubated 10 min at 25°C, and were stopped by the addition of 1 ml Biomol Green Reagent (Enzo). Released of phosphate was determined by measuring the absorbance at 620 nm. The kinetic values were determined using the equation of *v* = *V*_max_ [S]/(*K*_m_ + [S]) where *v*, *V*_max_, *K*_m_ and [S] represent the initial velocity, the maximum velocity, the Michaelis constant and the substrate concentration, respectively. The *K*_cat_ was calculated by dividing *V*_max_ by the concentration of enzyme used in the reaction (*K*_cat_ = *V*_max_/[enzyme]).

### cAMP-binding assay

3′, 5′cAMP affinity matrix was purchased from Sigma. 4.5 mM of purified Clr-GST was incubated in batch with 200 μl of 3′, 5′cAMP-agarose, previously equilibrated in buffer A (100 mM sodium phosphate buffer [pH 7], 50 mM NaCl, at 4°C during 30 min on a rotary mixer. After washing 7 times with 1 ml buffer A, bound protein was eluted by 30 min incubation in 1 ml buffer A supplemented with 30 mM 3′, 5′cAMP or 30 mM 2′, 3′cAMP at 4°C. Fractions were analysed by 12% SDS-PAGE.

## Competing interests

The authors declared they have no competing interests.

## Authors’ contributions

CMD, VP and AMG collected and analysed data. AMG and JB directed the work. JB and CMB wrote and revised the manuscript. All authors have read and approved the final version of this manuscript.

## Supplementary Material

Additional file 1 **SpdA, a putative Class III phosphodiesterase.** (A) Phylogenetic tree generated with Phylogeny.fr [1]. The tree shows the phylogenetic relationship of the 15 IPR004843-containing proteins of *S. meliloti* with known phosphodiesterases from *M. tuberculosis* (Rv0805), *H. influenzae* (Icc) and *E. coli* (CpdA and CpdB). (B) Table showing the distribution of the five class III PDE subdomains among the 15 IPR004843-containing proteins from *S. meliloti*. Click here for file

Additional file 2 Plasmids used in this study. Click here for file

Additional file 3 **Molecules and conditions tested for expression of *spdA ex planta.***
Click here for file

Additional file 4 **Enzymatic characteristics of purified SpdA.** (A)Lineweaver-Burk representation of SpdA kinetics of hydrolysis of 2′, 3′ cAMP. Purified SpdA was assayed as described in methods. (B)SpdA kinetic values. Click here for file

Additional file 5 **SpdA does not require metal cofactor for 2′, 3′ cAMP hydrolysis.** (A) Activity assayed in absence (CT) or presence of ions chelators. (B) SpdA activity in absence (CT) or presence of added bivalent ions. Click here for file

Additional file 6 **2′, 3′ cAMP weakens *smc02178-lacZ *expression.** (A) *smc02178-lacZ* expression was monitored *ex planta* in *S.meliloti* 1021 WT and ΔSpdA background strains after addition of 2.5 mM 3′, 5′-cAMP and/or 7.5 mM 2′, 3′-cAMP. ***p < 1.3E-06, **p < 0.0001, *p < 0.003 with respect to the wild type. (B) *hemA-lacZ* expression was monitored *ex planta* in *S. meliloti* 1021 WT and ΔSpdA background strains after addition of 2.5 mM 3′, 5′-cAMP and/or 7.5 mM 2′, 3′-cAMP. Click here for file

Additional file 7 **Growth characteristics and stress adaptability of the ΔSpdA mutant.** (A) Growth curves of 1021 WT and ΔSpdA mutant strains in LBMC or in VGM supplemented or not with 7.5 mM 2′, 3′ cAMP. (B and C) sensitivity of 1021 WT and ΔSpdA strains to SDS (B) and heat shock (C) (see methods for details). Click here for file

Additional file 8 ***spdA* mutant symbiotic phenotype.** (A) Nodulation kinetics on *M. sativa* following inoculation with *S. meliloti* 1021 and ΔSpdA mutant. (B) Dry weight of *M. sativa* shoots 35 dpi (C and D). Expression pattern of the *smc02178-lacZ* reporter gene fusion in young (7dpi) nodules of *M. sativa* following inoculation with *S. meliloti* 1021 (C) and ΔSpdA mutant (D). Click here for file

Additional file 9 Bacterial strains used in this study. Click here for file

Additional file 10 Primers and oligonucleotides used in this work. Click here for file
