# Supplementary material for: Biochemical and functional characterization of SpdA, a 2′, 3′cyclic nucleotide phosphodiesterase from Sinorhizobium meliloti
Source: BMC Microbiol. 2013 Nov 26;13:268. doi: 10.1186/1471-2180-13-268 (PMC4222275; doi:10.1186/1471-2180-13-268)
Supplement: Additional file 1 — SpdA, a putative Class III phosphodiesterase. (A) Phylogenetic tree generated with Phylogeny.fr [1]. The tree shows the phylogenetic relationship of the 15 IPR004843-containing proteins of S. meliloti with known phosphodiesterases from M. tuberculosis (Rv0805), H. influenzae (Icc) and E. coli (CpdA and CpdB). (B) Table showing the distribution of the five class III PDE subdomains among the 15 IPR004843-containing proteins from S. meliloti. [file 1471-2180-13-268-S1.pdf]

A

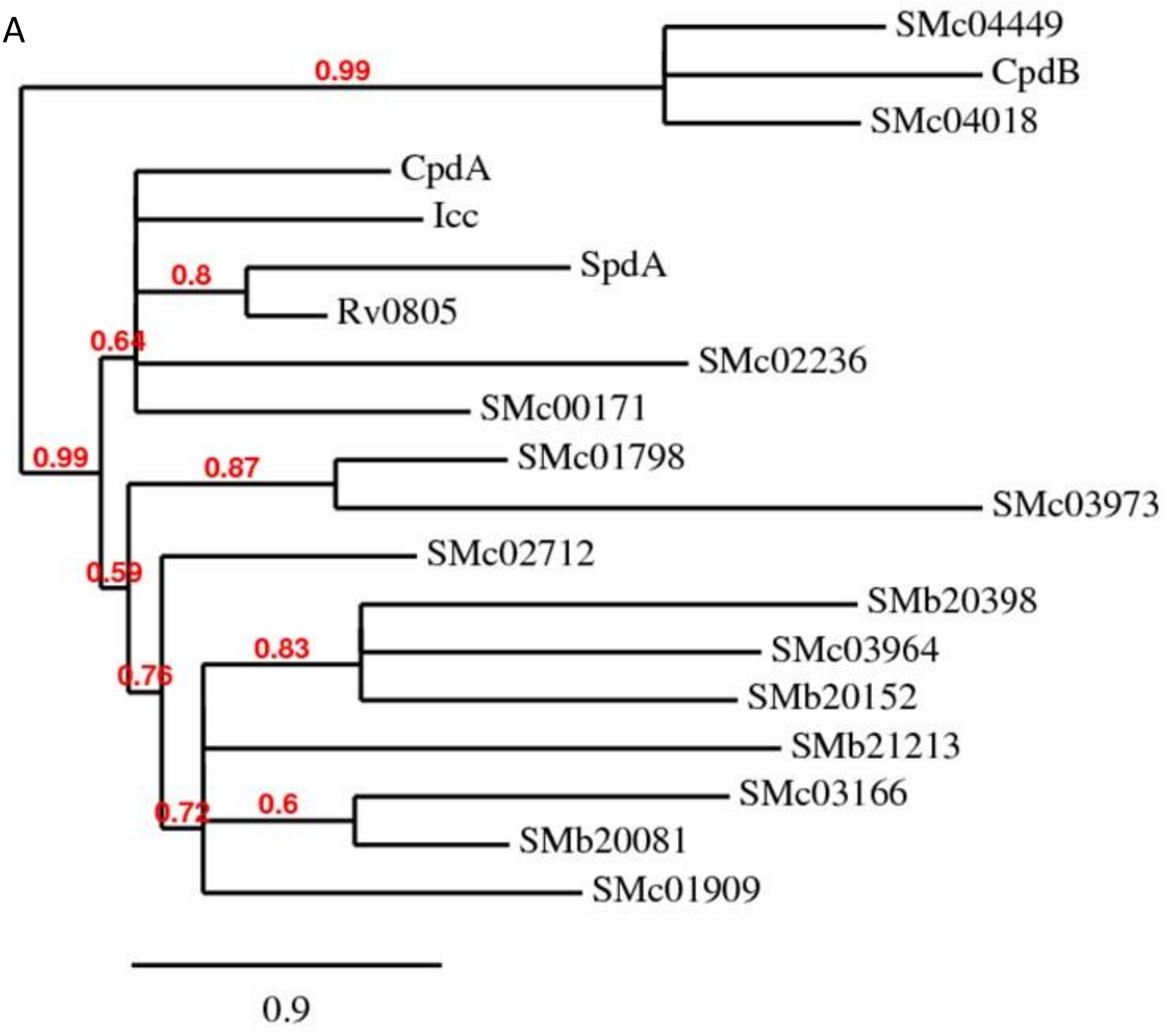

B

	I	II	III	IV	V
SpdA					
SMc02712					
SMb20081					
SMc01909					
SMc03166					
SMb20152					
SMc00171					
SMc01798					
SMc02236					
SMb21213					
SMc03964					
SMc04449					
SMc04018					
SMb20398					
SMc03973					

Additional file 1: **SpdA, a putative class III Phosphodiesterase.** (A) Phylogenetic tree generated with Phylogeny.fr [1]**.** The tree shows the phylogenetic relationship of the 15 IPR004843-containing proteins of *S. meliloti* with known phosphodiesterases from *M. tuberculosis* (Rv0805), *H. influenzae* (Icc) and *E. coli* (CpdA and CpdB). (B) Table showing the distribution of the five class III PDE subdomains among the 15 IPR004843-containing proteins from *S. meliloti.*

1. Dereeper A, Guignon V, Blanc G, Audic S, Buffet S, Chevenet F, Dufayard JF, Guindon S, Lefort V, Lescot M *et al*: **Phylogeny.fr: robust phylogenetic analysis for the non-specialist**. *Nucleic Acids Res* 2008, **36**(Web Server issue):W465-469.
