# Supplementary material for: Biochemical and functional characterization of SpdA, a 2′, 3′cyclic nucleotide phosphodiesterase from Sinorhizobium meliloti
Source: BMC Microbiol. 2013 Nov 26;13:268. doi: 10.1186/1471-2180-13-268 (PMC4222275; doi:10.1186/1471-2180-13-268)
Supplement: Additional file 2 — Plasmids used in this study. [file 1471-2180-13-268-S2.pdf]

Additional file 2. Plasmids used in this study

Plasmid	Description	Reference/source
pGEX-2T	Cloning vector containing a N-terminal GST Tag sequence, a Taq promoter and a lacIq coding sequence, Amp^r	GE Healthcare
pGEX ::clr	pGEX-2T cloning vector containing *clr* , Amp^r	This work
pET-22b(+)	Cloning vector containing a C-terminal His Tag sequence, a T7 promoter and a lacI coding sequence, Amp^r	Novagen
pET ::2179	pET-22b(+) cloning vector containing *spdA,* Amp^r	This work
pGD926	pRK290 derivative containing a promoterless *lacZ* gene, Tet^r	[1]
pGD2178	pGD926 containing the 399 bp promoter region upstream ATG of the *smc02178* gene, Tet^r	[2]
pAMG2178	pGD926 containing the 102 bp promoter region upstream ATG of the *smc02178* gene, Tet^r	This work
pAMG2178ΔClrbox	pGD926 containing the 102 bp promoter region deleted for the Clr box upstream ATG, Tet^r	This work
pGD2179	pGD926 containing the 122 bp promoter region upstream ATG of the *spdA* gene , Tet^r	This work
pXLGD4	hemA-lacZ reporter plasmid, Tet^r	[3]
pCM351	Allelic exchange vector bearing a gentamicin cassette flanked by loxP sites, Gen^r, Tet^r	[4]
pRK600	Helper conjugative plasmid, ColE1 replicon with RK2 transfer region, Chl^r	[5]

1. Ditta G, Schmidhauser T, Yakobson E, Lu P, Liang XW, Finlay DR, Guiney D, Helinski DR: **Plasmids related to the broad host range vector, pRK290, useful for gene cloning and for monitoring gene expression**. *Plasmid* 1985, **13**(2):149-153.
2. Tian CF, Garnerone AM, Mathieu-Demazière C, Masson-Boivin C, Batut J: **Plant-activated bacterial receptor adenylate cyclases modulate epidermal infection in the Sinorhizobium meliloti-Medicago symbiosis**. *Proc Natl Acad Sci U S A* 2012, **109**(17):6751-6756.
3. Leong SA, Williams PH, Ditta GS: **Analysis of the 5' regulatory region of the gene for delta-aminolevulinic acid synthetase of Rhizobium meliloti**. *Nucleic Acids Res* 1985, **13**(16):5965-5976.
4. Marx CJ, Lidstrom ME: **Broad-host-range cre-lox system for antibiotic marker recycling in gram-negative bacteria**. *Biotechniques* 2002, **33**(5):1062-1067.
5. Finan TM, Kunkel B, De Vos GF, Signer ER: **Second symbiotic megaplasmid in Rhizobium meliloti carrying exopolysaccharide and thiamine synthesis genes**. *J Bacteriol* 1986, **167**(1):66-72.
