# Supplementary material for: Biochemical and functional characterization of SpdA, a 2′, 3′cyclic nucleotide phosphodiesterase from Sinorhizobium meliloti
Source: BMC Microbiol. 2013 Nov 26;13:268. doi: 10.1186/1471-2180-13-268 (PMC4222275; doi:10.1186/1471-2180-13-268)
Supplement: Additional file 3 — Molecules and conditions tested for expression of spdA ex planta. [file 1471-2180-13-268-S3.pdf]

Additional file 3. Molecules and conditions tested for expression of *spdA ex planta*

3'5'cAMP 5 mM	Proline 0,2% (v/v)
2'3'cAMP 5 mM	$MnSO_4$ 1 mM
3'5'cGMP 5 mM	Microoxic growth conditions
5'AMP 5 mM	Growth temperature 22°C
M. sativa shoots extract 10% (v/v)	$FeCl_3$ deficiency (0,35 µM)
M. sativa nodules extract 10% (v/v)	$FeCl_2$ 35 µM
M. sativa flowers extract 10% (v/v)	$MnCl_2$ 35 µM
M. trucatula roots exudates 10% (v/v)	Spermine NONOate 25 µM
M. truncatula seeds exudates 10% (v/v)	H_2O_2 5 mM
Luteoline 10 µM	
