# Supplementary material for: Biochemical and functional characterization of SpdA, a 2′, 3′cyclic nucleotide phosphodiesterase from Sinorhizobium meliloti
Source: BMC Microbiol. 2013 Nov 26;13:268. doi: 10.1186/1471-2180-13-268 (PMC4222275; doi:10.1186/1471-2180-13-268)
Supplement: Additional file 4 — Enzymatic characteristics of purified SpdA. (A)Lineweaver-Burk representation of SpdA kinetics of hydrolysis of 2′, 3′ cAMP. Purified SpdA was assayed as described in methods. (B)SpdA kinetic values. [file 1471-2180-13-268-S4.pdf]

A

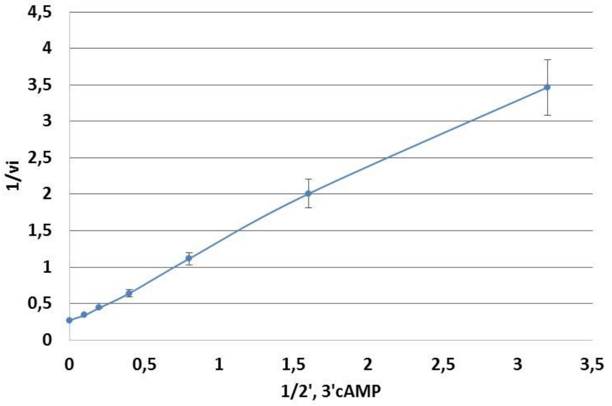

B

Vmax (µmole.min^{-1}.mg^{-1})	Km (mM)	KCat (S^{-1})	KCat/Km (M^{-1} S^{-1})
3.79 (+/- 1.36)	3.84 (+/- 1.05)	2 (+/- 0.7)	520

<u>**Additional file 4**</u>: **Enzymatic characteristics of purified SpdA.** (A) Lineweaver-Burk representation of SpdA kinetics of hydrolysis of 2', 3'cAMP. Purified SpdA was assayed as described in methods. (B) SpdA kinetic values.
