# Supplementary material for: Biochemical and functional characterization of SpdA, a 2′, 3′cyclic nucleotide phosphodiesterase from Sinorhizobium meliloti
Source: BMC Microbiol. 2013 Nov 26;13:268. doi: 10.1186/1471-2180-13-268 (PMC4222275; doi:10.1186/1471-2180-13-268)
Supplement: Additional file 5 — SpdA does not require metal cofactor for 2′, 3′ cAMP hydrolysis. (A) Activity assayed in absence (CT) or presence of ions chelators. (B) SpdA activity in absence (CT) or presence of added bivalent ions. [file 1471-2180-13-268-S5.pdf]

A

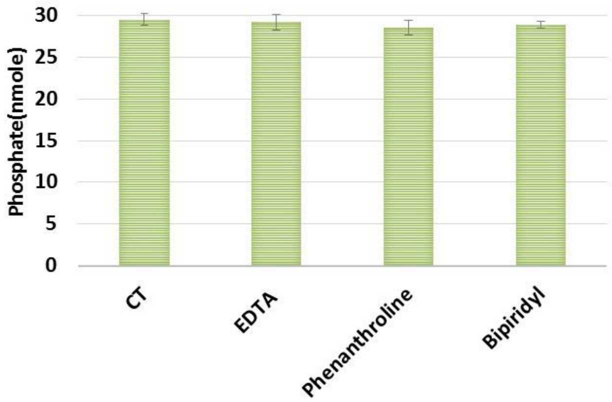

B

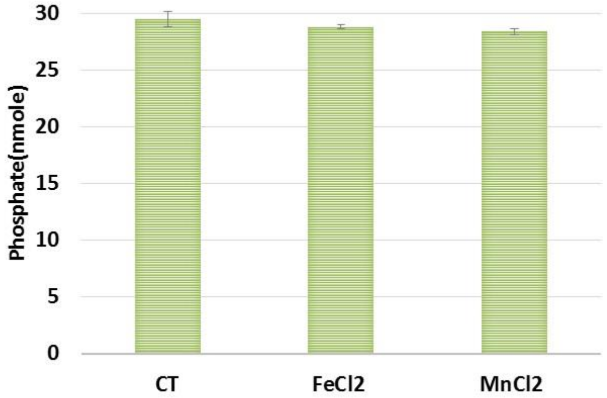

<u>Additional file 5:</u> **SpdA does not require metal cofactor for 2', 3'cAMP hydrolysis.** (A) Activity assayed in absence (CT) or presence of ions chelators. (B) SpdA activity in absence (CT) or presence of added bivalent ions.
