# Supplementary material for: Biochemical and functional characterization of SpdA, a 2′, 3′cyclic nucleotide phosphodiesterase from Sinorhizobium meliloti
Source: BMC Microbiol. 2013 Nov 26;13:268. doi: 10.1186/1471-2180-13-268 (PMC4222275; doi:10.1186/1471-2180-13-268)
Supplement: Additional file 6 — 2′, 3′ cAMP weakens smc02178-lacZ expression. (A) smc02178-lacZ expression was monitored ex planta in S.meliloti 1021 WT and ΔSpdA background strains after addition of 2.5 mM 3′, 5′-cAMP and/or 7.5 mM 2′, 3′-cAMP. ***p < 1.3E-06, **p < 0.0001, *p < 0.003 with respect to the wild type. (B) hemA-lacZ expression was monitored ex planta in S. meliloti 1021 WT and ΔSpdA background strains after addition of 2.5 mM 3′, 5′-cAMP and/or 7.5 mM 2′, 3′-cAMP. [file 1471-2180-13-268-S6.pdf]

A

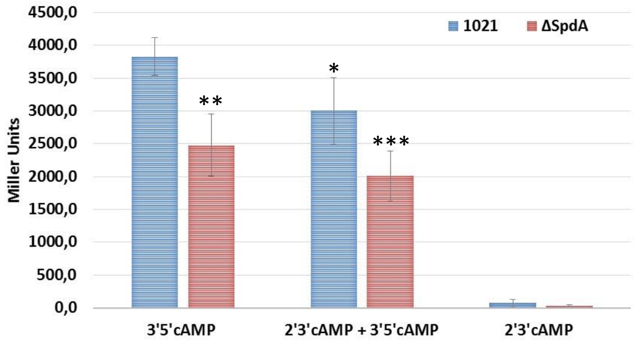

B

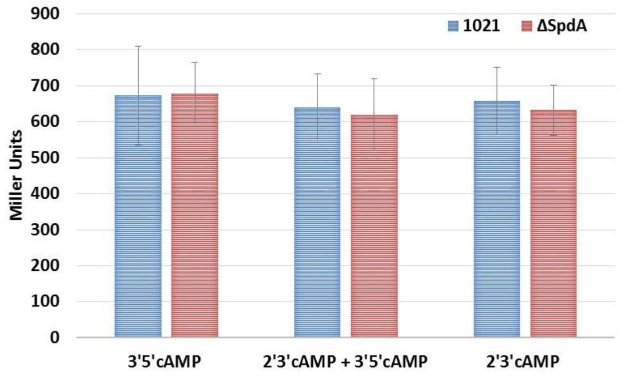

<u>Additional file 6:</u> 2', 3'cAMP weakens *smc02178-lacZ* expression. (A) *smc02178-lacZ* expression was monitored *ex planta* in *S. meliloti* 1021 WT and ΔSpdA background strains after addition of 2.5 mM 3', 5'-cAMP and/or 7.5 mM 2', 3'-cAMP. ***p<1.3E-06, **p<0.0001, *p<0.003 with respect to the wild type.(B) *hemA-lacZ* expression was monitored *ex planta* in *S. meliloti* 1021 WT and ΔSpdA background strains after addition of 2.5 mM 3', 5'-cAMP and/or 7.5 mM 2', 3'-cAMP.
