# Supplementary material for: Biochemical and functional characterization of SpdA, a 2′, 3′cyclic nucleotide phosphodiesterase from Sinorhizobium meliloti
Source: BMC Microbiol. 2013 Nov 26;13:268. doi: 10.1186/1471-2180-13-268 (PMC4222275; doi:10.1186/1471-2180-13-268)
Supplement: Additional file 7 — Growth characteristics and stress adaptability of the ΔSpdA mutant. (A) Growth curves of 1021 WT and ΔSpdA mutant strains in LBMC or in VGM supplemented or not with 7.5 mM 2′, 3′ cAMP. (B and C) sensitivity of 1021 WT and ΔSpdA strains to SDS (B) and heat shock (C) (see methods for details). [file 1471-2180-13-268-S7.pdf]

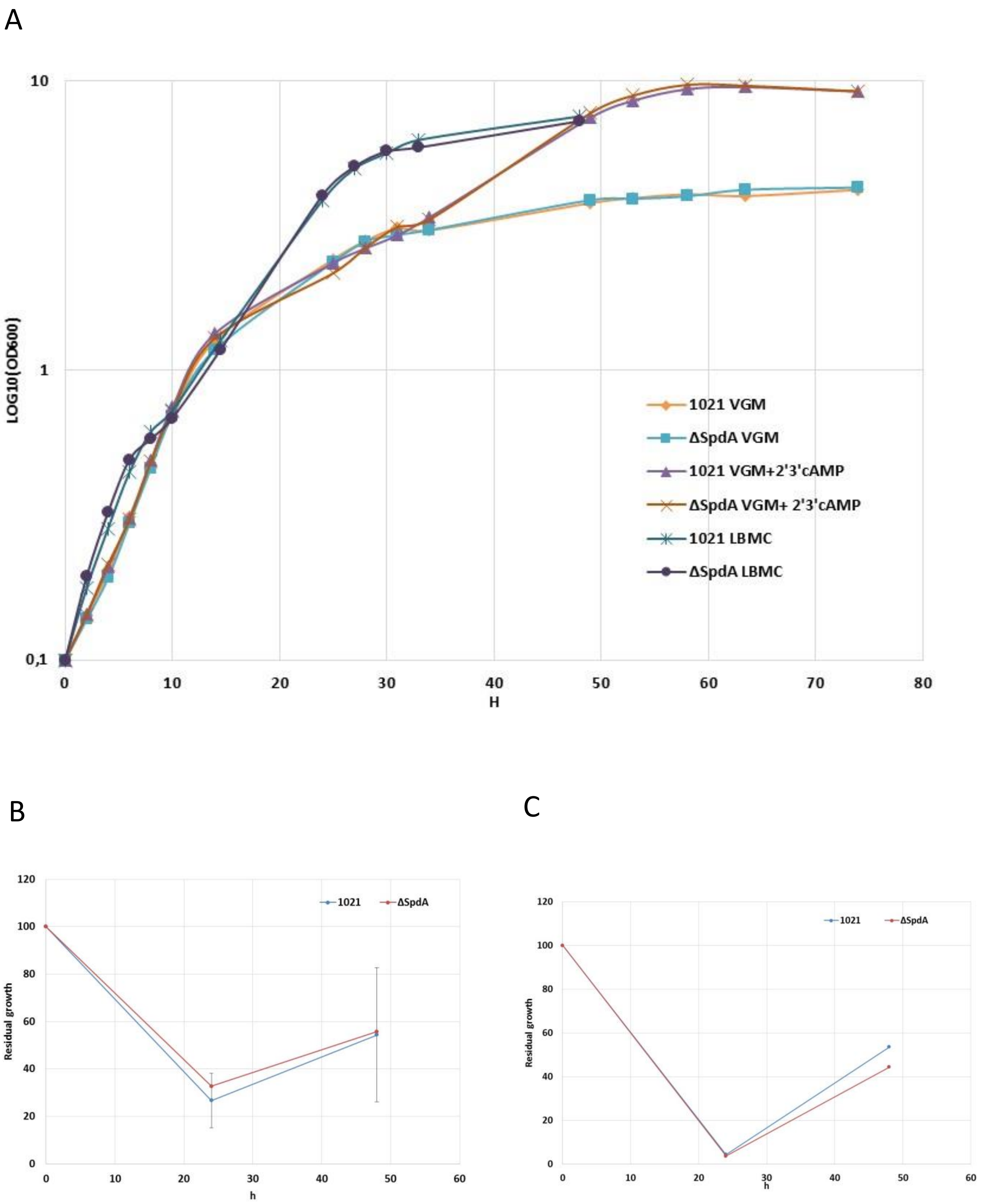

<u>Additional file 7:</u> Growth characteristics and stress adaptability of the ΔSpdA mutant. (A) Growth curves of 1021 WT and ΔSpdA mutant strains in LBMC or in VGM supplemented or not with 7.5 mM 2', 3'cAMP. (B and C) sensitivity of 1021 WT and ΔSpdA strains to SDS (B) and heat shock (C) (see methods for details).
