# Supplementary material for: Biochemical and functional characterization of SpdA, a 2′, 3′cyclic nucleotide phosphodiesterase from Sinorhizobium meliloti
Source: BMC Microbiol. 2013 Nov 26;13:268. doi: 10.1186/1471-2180-13-268 (PMC4222275; doi:10.1186/1471-2180-13-268)
Supplement: Additional file 8 — spdA mutant symbiotic phenotype. (A) Nodulation kinetics on M. sativa following inoculation with S. meliloti 1021 and ΔSpdA mutant. (B) Dry weight of M. sativa shoots 35 dpi (C and D). Expression pattern of the smc02178-lacZ reporter gene fusion in young (7dpi) nodules of M. sativa following inoculation with S. meliloti 1021 (C) and ΔSpdA mutant (D). [file 1471-2180-13-268-S8.pdf]

A

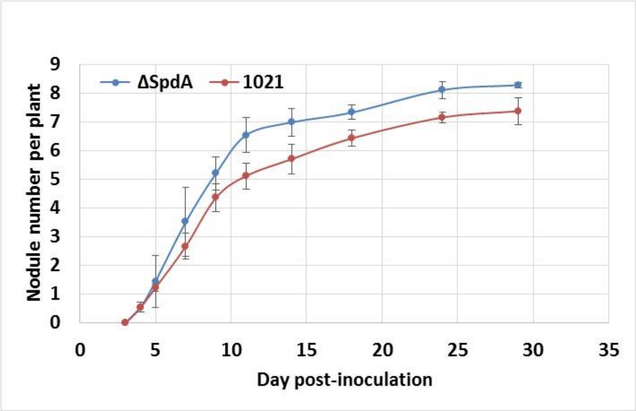


B

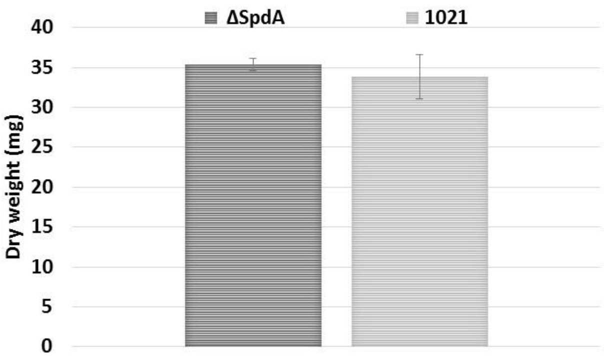


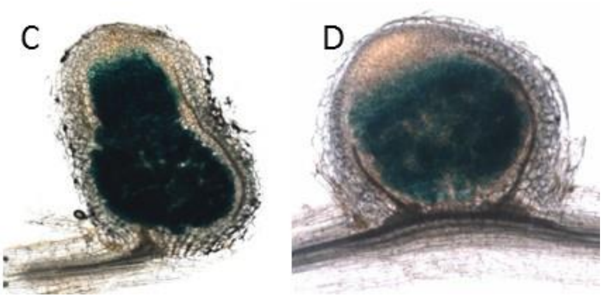


**<u>Additional file 8: *spdA*</u> mutant symbiotic phenotype.** (A) Nodulation kinetics on *M. sativa* following inoculation with *S. meliloti* 1021 and ΔSpdA mutant. (B) Dry weight of *M. sativa* shoots 35 dpi (C and D). Expression pattern of the *smc02178-lacZ* reporter gene fusion in young (7dpi) nodules of *M. sativa* following inoculation with *S. meliloti* 1021 (C) and ΔSpdA mutant (D).
