# Supplementary material for: Biochemical and functional characterization of SpdA, a 2′, 3′cyclic nucleotide phosphodiesterase from Sinorhizobium meliloti
Source: BMC Microbiol. 2013 Nov 26;13:268. doi: 10.1186/1471-2180-13-268 (PMC4222275; doi:10.1186/1471-2180-13-268)
Supplement: Additional file 9 — Bacterial strains used in this study. [file 1471-2180-13-268-S9.pdf]

Additional file 9. Bacterial strains used in this study

Strain	Description	Reference/Source
1021	Str^{r} derivative of S. meliloti strain SU47	[1]
GMI11567	1021 *clr*::pVO155, Str^{r}, Neo^{r}	[2]
GMI11892	1021 *ΔspdA*, Str^{r}, Gen^{r}	This work
SP850	*E. coli cyaA1400* Amp^{r}, Kan^{r}	[3]
DH5α	*E. coli fhuA2 Δ(argF-lacZ)U1 69 phoA glnV44 Φ80 Δ(lacZ)M15 gyrA96 recA1 relA1 endA1 thi-1 hsdR17*	Bethesda Research
BL21(DE3)	F^{-} ompT gal dcm lon hsdS$_{B}$(r$_{B}^{-}$ m$_{B}^{-}$) λ(DE3) pLysS(cm^{r})	Stratagene

1. Meade HM, Long SR, Ruvkun GB, Brown SE, Ausubel FM: **Physical and genetic characterization of symbiotic and auxotrophic mutants of Rhizobium meliloti induced by transposon Tn5 mutagenesis**. *J Bacteriol* 1982, **149**(1):114-122.
2. Tian CF, Garnerone AM, Mathieu-Demazière C, Masson-Boivin C, Batut J: **Plant-activated bacterial receptor adenylate cyclases modulate epidermal infection in the Sinorhizobium meliloti-Medicago symbiosis**. *Proc Natl Acad Sci U S A* 2012, **109**(17):6751-6756.
3. Shah S, Peterkofsky A: **Characterization and generation of Escherichia coli adenylate cyclase deletion mutants**. *J Bacteriol* 1991, **173**(10):3238-3242.
