# Supplementary material for: Biochemical and functional characterization of SpdA, a 2′, 3′cyclic nucleotide phosphodiesterase from Sinorhizobium meliloti
Source: BMC Microbiol. 2013 Nov 26;13:268. doi: 10.1186/1471-2180-13-268 (PMC4222275; doi:10.1186/1471-2180-13-268)
Supplement: Additional file 10 — Primers and oligonucleotides used in this work. [file 1471-2180-13-268-S10.pdf]

Additional file 10. Primers and oligonucleotides used in this work

Gene	Primer name	Sequence 5' to 3'	5' end restriction site
spdA	2179left	CCCAAGCTTGATTGCAGTGATGAGGG	HindIII
spdA	2179right	GCGGATCCACCATGTGGAGGTCGGAG	BamHI
spdA	CreLox 2179 up Left	CGAGCTCGAATTCGATCTCGAACGCAAC	SacI
spdA	CreLox 2179 up Right	TCCCCGCGGGGAGTGGAGGTCGGAGAAAATGA	SacII
spdA	2179 Down NcoI	CATGCCATGGGGCATTCCCTTCTCGATCTT	NcoI
spdA	2179 Down HincII	CCGGTCGACGATGGGGCGTATGTCGTAGA	HincII
spdA	LNdeI2179	CATATGACGAAGCTCATCATTTTC	NdeI
spdA	LRHindIII2179	AAGCTTCGCTCCCACCGCGTCGCGCCTT	HindIII
clr	ClrBamHI	CGGGATCCATGGCTGAAGTCAT	BamHI
clr	ClrEcoRI	GGAATTCTCAATCCTCCTCCGG	EcoRI
smc02178	BamHI 2178	CGGGATCCAGGCTCGATCCAACGTGTTTCTTC	BamHI
smc02178	Hind BoxL	CCAAGCTTGCTGCTTCAACTCACCGTTT	HindIII
smc02178	2178 H	CGAAGCTTCGTCAGGACATAATCCTTGTCGAG	HindIII
smc02178	BoxLpstI	TTTCTGCAGCGGTGAAGGCGATGAAAC	PstI
smc02178	X2178	GCTCTAGAAGCTTCTGTCCAGCCCTGT	XbaI
smc02178	BoxRpstI	AAACTGCAGATGCCGGGTCGGGGC	PstI
smc02178	WTN8-	GGCATCTGTTCCGCGGGAAACAGCGGTG	
smc02178	WTN8+	CACCGCTGTTTCCCGCGGAACAGATGCC	
smc02178	MN8-	GGCATCGCCACCGCGGGAGCTGGCGGTG	
smc02178	MN8+	CACCGCCAGCTCCCGCGGTGGCGATGCC	
